# Preparing clinicians to be site investigators in multicenter clinical trials: A training program at an academic medical center

**DOI:** 10.1017/cts.2023.587

**Published:** 2023-07-10

**Authors:** Tammy L. Loucks, Diana Lee-Chavarria, Jillian Harvey, Rechelle Paranal, Signe Denmark, Patrick A. Flume, Marc Chimowitz, Tanya N. Turan

**Affiliations:** 1 South Carolina Clinical and Translational Research Institute, Medical University of South Carolina, Charleston, SC, USA; 2 Academic Affairs Faculty and Department of Obstetrics and Gynecology, Medical University of South Carolina, Charleston, SC, USA; 3 Department of Health Care Leadership and Management, College of Health Professions, Medical University of South Carolina, Charleston, SC, USA; 4 Office of Clinical Research, Medical University of South Carolina, Charleston, SC, USA; 5 Division of Pulmonary and Critical Care Medicine, Department of Medicine, College of Medicine, Medical University of South Carolina, Charleston, SC, USA; 6 Department of Neurology, College of Medicine, Medical University of South Carolina, Charleston, SC, USA

**Keywords:** Education, program, research personnel, clinical trial, capacity building

## Abstract

Clinical trials are essential in the translation of biomedical discoveries to new clinical interventions and therapeutics. Successful multisite clinical trials require qualified site investigators with an understanding of the full spectrum of processes and requirements from trial identification through closeout. New site investigators may be deterred by competing demands on their time, the complexity of administrative and regulatory processes for trial initiation and conduct, and limited access to experienced mentor networks. We established a Clinical Trialist Training Program (CTTP) and complimentary Clinical Trials Bootcamp at our institution to address these barriers and increase the number of local site investigators enabled to lead successful clinical trials. An initial cohort of four CTTP scholars received salary support with protected time, didactic training, assistance with study identification and start-up navigation, and quarterly progress meetings. By the end of the 12-month program, this initial cohort identified 33 new trials, utilized feasibility assessments, and reported being on target to sustain their protected time from new clinical trials. Bootcamp attendees demonstrated increased knowledge of resources, offices, and processes associated with clinical trial conduct. Our results support providing compensated protected time, training, and access to experienced clinical research professionals to enable clinicians to become successful site investigators.

## Introduction

Clinical trials are fundamental to the continuum of translational research conducted at academic medical centers. They provide patients with opportunities to participate in the development of novel therapeutics and diagnostics as well as access to potentially life-saving treatments or interventions before they are generally available [1]. Multisite clinical trials provide academic and leadership opportunities for local site investigators [2,3]. A cadre of qualified site investigators, representing the range of clinical specialties, are necessary to sustain and advance an institution’s comprehensive clinical trial portfolio.

Successful clinical trial programs require dedicated investigator effort to develop. Building a sustainable trial portfolio takes time and investigators must learn how to (1) identify new trial opportunities, (2) assess study feasibility by evaluating fit with their research and clinical interests and patient population, (3) evaluate resources needed to conduct study activities, (4) capture trial activity costs in budgets and contracts, and (5) develop their study team. In addition, efficient study start-up processes and planning for optimal participant recruitment and retention are needed to minimize the risk of low-enrolling and uninformative trials. Investments in these activities can help mitigate many of the challenges associated with clinical trials, including low or inefficient enrollment, and lengthy start-up times [4–7].

We conceived the concept of a Clinical Trialist Training Program (CTTP) based on the experience of partnering with two physician investigators to establish their clinical trial portfolios. The investigators had not previously served as site principal investigators, and their respective departments had few clinical trials in their research portfolios. The Clinical and Translational Science Award (CTSA) trial innovation unit staff partnered with these two investigators to identify trials, assess feasibility, develop budgets, and navigate site initiation processes. This collaborative approach proved successful as multiple new clinical trials were initiated and each investigator went on to manage a successful trial portfolio [8].

Through this process, we recognized new site trial investigators faced challenges with identifying responsible institutional offices, understanding workflows, and locating resources to efficiently initiate new clinical trials in the complex environment of an academic medical institution. Also, the current physician reimbursement model based on clinical productivity could deter clinicians from engaging in such academic activities. We determined that potential new site investigators would benefit from a structured training program; however, any program would need to include sufficient protected time to develop the expertise to be effective site investigators and establish a successful clinical trial portfolio. Reinforcing this notion were the results from a recent research education needs-and-utilization survey where faculty at our institution identified time as a primary barrier to engaging in research training and development activities [9].

The CTTP and a Clinical Trials Bootcamp were subsequently developed to address these barriers and establish a pathway for new clinical investigators to become successful site trialists. The CTTP is a 12-month program to train clinicians to be effective site principal investigators for multisite clinical trials by providing compensated effort for one year and training and resources to support study identification, feasibility assessments, initiation, and management of new clinical trials. These new trials provide a foundation for the CTTP scholar to build a portfolio of impactful clinical trials that will generate funding to support their continued research effort, their study team, and associated research costs. Clinical Trials Bootcamp is an 8-hour program for clinicians that provides high-level information about study selection, initiation, approval, oversight, and conduct. We describe the implementation and initial outcomes of these two programs to build research capacity and develop new site clinical trialists.

## Methods

This activity is considered program development and not human subjects research and thus no institutional review board approval was required.

### Institutional Commitment

To ensure that clinicians would have adequate time to devote to the CTTP program, CTSA leaders secured commitment from the Dean of the College of Medicine including full salary and fringe benefits commensurate with ten percent effort for a 12-month period for up to four clinical investigators annually. Two of the four slots were specifically allocated to cancer center investigators. The CTSA has developed collaborative relationships with institutional offices overseeing clinical research and frequently serves to coordinate efforts across offices to address research barriers and streamline processes [10–15]. The Offices of Clinical Research, Research Integrity, and Research and Sponsored Programs contributed subject matter experts to develop content and participate in the Bootcamp and CTTP training activities.

### CTTP Recruitment, Review, and Selection

The trial innovation unit and the CTSA Translational Workforce Development program partnered to create and administer a request for applications and market the CTTP opportunity to all faculty in the College of Medicine. The request for applications was released during the last quarter of the calendar year and review and selection of candidates was completed by the end of the first quarter of the subsequent calendar year. This schedule allowed departments to budget a reduced clinical effort for the selected candidates beginning at the start of the new fiscal year (i.e., July 1).

Full-time faculty at or below the rank of Associate Professor with a clinical doctoral degree, board certification in their specialty, and limited previous experience leading clinical trials were eligible to apply. In addition, applicants were required to have an appointment with the institution’s cancer center to qualify for one of the two cancer center positions. Established investigators and investigators primarily seeking support to design investigator-initiated trials were not eligible.

Each applicant was required to provide a current curriculum vita and letter of interest describing their current clinical commitment, academic responsibilities for teaching, research, and administration, current and prior involvement in conducting clinical trials, level of interest for becoming a site trialist, research interests, access to patient population for research, and long-term career goals related to planning and conducting clinical trials. A letter of support from the applicant’s department chair was also required. The chair’s letter outlined departmental factors for consideration including research direction, current clinical trial portfolio, gaps in the portfolio that the applicant would help fill, interest in expanding the clinical trials portfolio, availability of senior site trialists for mentorship, and other resources available to support the applicant. Chairs were also required to include a statement committing to protect ten percent of the applicant’s time with a description of how the applicant’s clinical and other commitments would be modified if selected for the CTTP. All applications and letters were submitted to a secure central grant application portal for review.

Applications were initially screened by the CTTP program directors who selected the top candidates based on the criteria described above. These applicants were invited to a panel interview to evaluate their credentials, previous clinical trials experience, and future trial plans in more detail. The interview panels included the CTTP program directors and one to three additional faculty reviewers with relevant expertise. Panel members recused themselves if there were any conflicts of interest with the candidates. During the interview, candidates were asked to describe what they would like to gain from participation in the CTTP and how the program would help them meet their career goals. Specific discussion points were sent in advance for the candidates to consider. The interviewers ranked each candidate, and the two candidates with the highest scores from the College of Medicine and two from the cancer center were selected for the program.

### Bootcamp

The Clinical Trials Bootcamp was marketed broadly to clinical departments through the College of Medicine and CTSA newsletters and faculty emails. Participation was limited to faculty, and all new site investigators and experienced clinical trialists who desired a refresher were encouraged to attend. Registration via REDCap [16] was required to gain access to the virtual meeting platform. The opportunity was announced four months in advance so that clinicians would have time to arrange for clinical coverage to attend.

### Program Design

The overall design and content of the CTTP and Clinical Trials Bootcamp were informed by feedback from investigators, program and clinical trial leaders, and subject matter experts from the CTSA and institutional research support offices. Investigators who had worked with the trial innovation unit team to initiate new clinical trials were queried about what they considered most relevant for new investigators to know as new trialists. In addition to protected time, suggestions included introductions to and descriptions of relevant research resource offices, investigator and study team roles and responsibilities, the processes for study initiation, protocol review and budgeting, and effort determination.

Next, program leaders and subject matter experts from the CTSA and institutional research support offices reviewed these recommendations and added insights from their expertise and experiences on areas where new investigators experienced challenges in the study initiation and management processes. They also identified practices followed by successful clinical trial programs including evaluating feasibility, identifying the right trials for investigator interest, patient populations, and clinical resource requirements. Our approach was also informed by the framework developed by the Joint Task Force for Clinical Trial Competency with attention to the domains clinical study operations (4), study and site management (5), leadership and professionalism (7), and communications (8) [17]. The final CTTP and Clinical Trials Bootcamp activities and content incorporated the perspectives of multiple stakeholders representing nascent and seasoned investigators and the institutional offices overseeing clinical trials.

### Clinical Trialist Training Program (CTTP)

**Figure 1.**
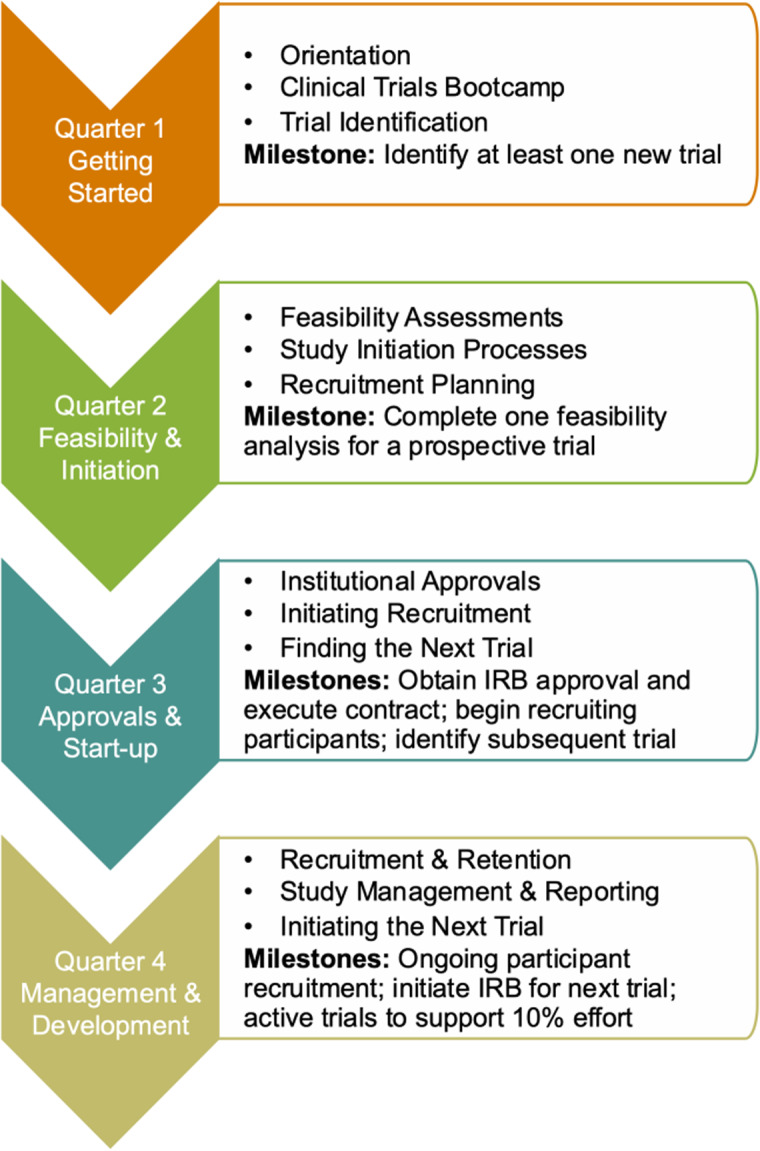
Clinical Trialist Training Program (CTTP) quarterly activities and milestones.

Program activities covered four main areas of study conduct (Fig. 1). Immediately following their notice of acceptance into the program trial innovation unit staff reached out to the scholars and began working with them to identify potential trials. trial innovation unit staff and CTTP Leaders acted as “navigators” providing one-on-one support to help participants achieve program milestones. During the Getting Started (Q1) phase, CTTP scholars took part in the Clinical Trials Bootcamp, were oriented to the program, and charged with identifying at least one new trial. At orientation participants met with members of the trial innovation unit and institutional research support offices who would work with them throughout their appointments to identify and implement new clinical trials. Participants met individually at the end of each quarter with CTTP leaders and trial innovation unit staff to report on progress, discuss challenges and concerns, and strategize course corrections as necessary to achieve their goals.

During the Feasibility and Initiation phase (Q2) participants were introduced to resources and approaches to conducting feasibility assessments and the presite visit process. The milestone for second quarter was to complete at least one feasibility assessment. These assessments encompassed the potential for recruitment of participants, resource, and personnel requirements for study conduct and budgetary considerations. During the Approvals and Start-up phase (Q3), participants worked to obtain all institutional approvals such as human subjects approvals, prospective reimbursement analysis for research billing compliance, budget development and contractual agreements for their primary study, and begin recruiting study participants. They also continued to work with the trial innovation unit team to identify subsequent trials to initiate. In the last quarter of the program, participants focused on study management and the development of their trial portfolios for sustainability. Participants also met quarterly with CTTP leadership and trial innovation unit staff to review progress, discuss any barriers to progress, and develop strategies to achieve program milestone.

### Clinical Trials Bootcamp

The Bootcamp program was delivered during two half-day virtual sessions and provided a high-level overview of key topics related to clinical trial identification, initiation, and management including a review of investigator roles and responsibilities and study team development, see supplemental material for a full list of topics. Institutional offices with regulatory and oversight responsibilities for clinical trials were introduced and resources to enable corporate clinical trials were also presented. Breaks were incorporated to promote interaction between attendees and presenters, address questions, and share strategies to overcome common challenges.

### Evaluation Approach

The CTTP program evaluation encompassed a two-prong formative and summative approach: developing mechanisms to track and manage CTTP scholar progress toward goals and milestones and of the overall program’s implementation impact. To quantify new sponsored research awards to participating CTTP scholars, we examined our institutional award database for the two years prior and during the one-year program.

### CTTP Participant Needs Assessment

A pre-program survey using REDCap [16,18] was implemented to assess the individual needs of each CTTP scholar. The information gathered informed program leadership, administrators, and trial innovation unit staff how to tailor their approach to each respective CTTP scholar and their identified study needs. This approach allowed for program administrators to adapt their interventions based on the individual trial’s changing needs and respond to any emergent issues that might arise from the study sponsor or the general research environment.

### CTTP Program Impact Assessment

A post-program survey using REDCap [16,18] was implemented to track the individual scholar’s progress, attain feedback about their experience with the CTTP, and glean insights for improvement. A qualitative exit interview was administered using Microsoft Teams for efficient transcription. Using this programmatic feedback, recommendations can be developed on how best to adapt the program to meet future scholar needs. Additionally, scholar progress and study related outputs can be tracked in our Research Integrated Network of Systems (RINS) [13].

### Bootcamp

All participants were invited to complete a brief ten question evaluation and provide feedback on the overall program. In addition, participants were asked to complete a second questionnaire about their level of knowledge of research processes and offices before and after attending the two-day program. The evaluation and knowledge questionnaires were administered using REDCap [16] and have been provided as supplemental materials. Attendees received a link to a shared folder in Box upon submission of their evaluation. The folder included copies of the presentations and supplemental resources relevant to the office or topic area presented.

## Results

### CTTP

Eleven applications were received for the inaugural CTTP cohort, the four positions were filled and included two faculty at the Assistant Professor and two at the Associate Professor ranks. Two CTTP participants were women, three identified as White and one identified as more than one race. Selected investigators represented gynecologic oncology (*n* = 2), cardiothoracic (*n* = 1), and pediatrics specialties (*n* = 1). Progress toward quarterly milestones was excellent for the inaugural CTTP cohort with all four participants achieving 100% of milestones; specific details for these milestones are presented in Table 1.

**Table 1.** Progress toward program milestones reported by Clinical Trialist Training Program (CTTP) scholars. progress may carry from one quarter to others

Quarter/Milestone	Milestone indicators	Activities reported starting			
		Q1	Q2	Q3	Q4
Quarter 1. Identify one new trial	New Trials Identified	18	6	6	3
	Confidential Disclosure Agreements	4	3	1	0
Quarter 2. Complete one feasibility analysis	Feasibility/Financial Assessments	10	3	1	1
	Pre-Selection Visits	4	1	3	0
	New Studies Initiated for start-up (i.e., institutional review processes initiated)	1	5	2	0
	Developing Recruitment Strategies	6	9	4	9
Quarter 3. Obtain one IRB approval Execute contract Begin recruiting Identify next trial	IRB Approvals	1	1	0	0
	Study Site Selected	4	1	0	7
	Studies Pending Contracts	1	1	1	0
	Studies in Startup	1	1	0	7
	New Trial Open to Accrual	1	1	1	3
	Studies Enrolling	1	1	6	9
	Site Initiation Visits	2	1	1	0
Quarter 4. IRB approval for next trial Ongoing recruitment Trials to support 10% effort	Subsequent Trials in Pipeline to Initiate	1	5	4	0
	Studies with Ongoing Recruitment	1	1	6	9
	Closed Studies	0	0	0	2

The scholars reported identifying a total of 33 new trials; of those pursued each was accompanied by a feasibility assessment. From the institutional award database, we documented nine awards totaling $2.2M for the scholars during the 12-month CTTP. These included seven clinical trials of which five were corporate sponsored and two were funded through philanthropic sources. Three CTTP scholars had received four new clinical trial awards (3 corporate and 1 foundation) as primary investigator in the 24 months preceding their entry into the CTTP program. Together these four awards totaled $509,815.

Best practices introduced during CTTP were also noted in the exit interview; see Table 2 for selected quotes. Specifically, one scholar had identified a new trial prior to beginning CTTP and later declined that trial based on a feasibility assessment they completed as part of CTTP.

**Table 2.** Selected quotes from Clinical Trialist Training Program (CTTP) scholars provided during their exit interviews

Area	CTTP scholar quote
Portfolio Development	*“So, we’re building a nice portfolio for the department. Last year we had one patient on trial and by the end of this year we’ll have 10…a significant increase in trial number.”* *“I’ve been able to get more trials open in areas where we have need for that and I've been able to focus a little bit more on offering patients these trials, being able to speak to them a little bit more in clinic about them and then get them screened and enrolled.”*
Feasibility Assessment	*“CTTP helped me figure out that… we just need to cut our losses, like we had actually gone through a feasibility and was site selected.”*
Sustainability	*“I will be very close to 10% [investigator salary support], but I think it’s probably in the upper single digit percentiles when I put it all together.”* *“…even though I have all these studies and all this other stuff, it doesn't equal to 10% [investigator salary support] right away, right? It’s always in the future.”*
Satisfaction	*“I would need about three more years to be where I'm at today, so it definitely accelerated my career trajectory.”*

A few of the challenges that emerged during the interviews were related to salary support (Table 2, Sustainability). None of the scholars had the full 10% salary support by the end of the program, though a percentage had been achieved. However, all the scholars anticipated having salary support at 10% once their study recruitment was underway and billing reimbursements were implemented and generating revenue. Suggested program modifications included extending the duration of support to two years with decreasing institutional support as new trials are initiated, adding focused didactic sessions on topics of relevance, and creating networking opportunities for CTTP participants.

Overall, three of the four participants said the program met their expectations and some identified it as exceeding expectations in certain areas. One participant found the program to be highly beneficial but rated the program lower indicating that they had hoped to receive additional training related to investigator-initiated federal and foundation processes. The participant acknowledged that they understood the focus of the program was on multisite clinical trials with per-patient reimbursement. During individual exit interviews, scholars indicated that they increased their departmental portfolios with their CTTP studies. They reported being able to offer their patients trial opportunities that had not been available prior to starting the CTTP program, and that the rate of their trial award success was accelerated by the supports received through the program. Suggestions for program enhancements included interspersing more didactics throughout the year to reinforce concepts, holding in-person cohort meetings, and incorporating discussions related to processes for non-industry sponsored trials. Overall, respondents felt that the program greatly enhanced their research careers and highlighted several components of the program as extremely helpful, including the bootcamp, quarterly progress meetings, and ongoing programmatic support.

### Bootcamp

There were 58 registrants for the program and 45 attended the sessions on day one and 39 attended on day two. Seventy-one percent of attendees completed the Bootcamp evaluation; 63% of those had not served as a principal investigator but 75% indicated that they had been involved in another capacity, primarily as a co-investigator. Nearly all participants (97%–100%) agreed or strongly agreed that the Bootcamp met their expectations, provided a good introduction and overview of the offices and resources supporting clinical trials and principal investigator responsibilities, was well organized, provided adequate time to the topics covered and opportunities for discussion, and would recommend attendance to a colleague. Most (97%) indicated they planned to pursue opportunities to conduct a trial. Several suggested including a mock study or case scenario in subsequent Bootcamps to track a new trial through the process.

**Figure 2.**
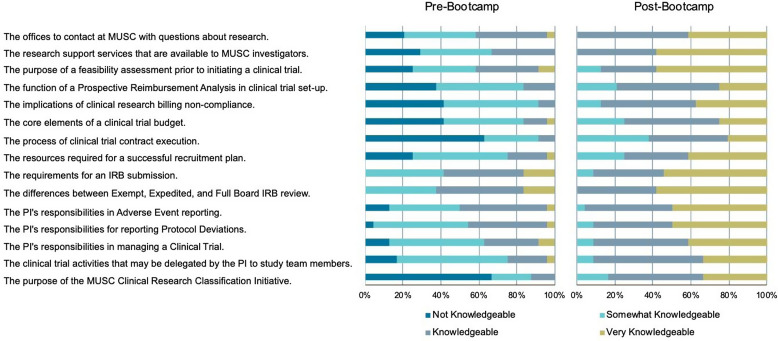
Ratings of knowledge about key research offices, resources, processes, and responsibilities associated with the conduct of clinical trials before (Pre-bootcamp) and after (Post-bootcamp), *N* = 24 participants. (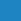 Not knowledgeable, 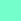 Somewhat knowledgeable, 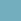 Knowledgeable, 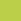 Very knowledgeable). MUSC = Medical University of South Carolina.

A total of 24 attendees completed the pre- and post- bootcamp knowledge questionnaires. For each of the fourteen areas, there were notable changes in the level of knowledge reported following the Bootcamp. These results are shown in Fig. 2.

## Discussion

Our approach to developing new and successful site trialists included compensated effort for one year as well as training and resources to support study identification, feasibility assessments, initiation, and management. All participants surpassed program milestone expectations for identifying new trials, conducting feasibility assessments, and opening new trials for recruitment.

A cornerstone of the program was an institutional commitment to developing effective site trialists. This included both salary support and pedagogical contributions from the CTSA program and institutional research offices to develop and deliver training and navigation support to CTTP scholars and bootcamp attendees. Salary support enabled clinicians in the CTTP program to reduce their clinical effort for time to identify new trials, develop knowledge and gain experience to successfully lead clinical trials; barriers reported by investigators who opted to discontinue conducting trials after their first experience [19]. The importance of this support was identified by participants during their exit interviews. One participant stated, *“[Salary Support] That was a huge appeal I think for me and critical component for me to be productive”* and another commented that the *“most helpful thing is that I got clinical buy-down.”*

The effort contributed by the CTSA program and institutional support offices was greater in the initial year to develop the program, educational presentations, evaluation tools, and other materials such as the request for applications, marketing materials, and a webpage. Ongoing leadership, coordination, and management of the combined CTTP and Bootcamp offerings are estimated to be in the range of 5%–10% for the program director and 10% for the program manager. Assistance with feasibility assessments, identification of new study opportunities, research navigation consultations, contract and budget development and negotiation are part of the services provided by the CTSA, and the research support offices at our institution and are not included in this estimate.

The CTTP has the long-term goal of developing site clinical trialists who can sustain a successful clinical trials program. Although not all participants achieved sufficient reimbursement for completed study activities to sustain 10% salary support once their participation in CTTP ended, they all reported that they were on target to have sufficient trial revenue to support their effort. Given accurate budgeting and sufficient enrollment, the CTTP scholars are predicted to have sustained success.

CTTP participants also noted during interviews that they were able to provide their patients more options for clinical trial participation. Given the time from trial identification to opening can take months [5], we were pleased with the scholars having 9 new awards recorded within the initial 12 months.

As a result of the program’s success, CTTP program leaders presented an interim progress update to senior leadership in the College of Medicine that resulted in continued support for another year of CTTP funding. Thirteen qualified clinicians applied for the 2022–2023 cohort and four were selected. In addition, two department chairs pledged departmental financial support for protected time for their interested faculty, allowing the CTTP to enroll six participants in Cohort 2.

Feedback from the initial CTTP cohort was used to enhance CTTP didactics, such as including more in-depth training in budgeting and planning, resulting in significant improvements to the program. Feedback from the current cohort will be used to further enhance the program as needed. Given the effectiveness of the CTTP, other CTSAs or institutions who wish to develop or expand their pool of site trialists and clinical trial portfolios may consider employing similar methods.

The process of initiating and managing clinical trials is complex and involves interactions with multiple institutional offices, research organizations, and sponsors; it also requires effort commitment to meet study milestones and reporting requirements, manage staff, and engage with participants as all while keeping abreast of the evolving regulatory environment. While CTTP provided an in-depth experience for four scholars, the Bootcamp served a larger pool of potential site investigators. Responses to the pre- and post- Bootcamp questionnaires indicate that attendees had a better understanding of the processes for finding, initiating, and managing a trial following the sessions. While we are not able to determine who among the attendees have pursued new trials, we hope that the information provided during Bootcamp prepared them to pick optimal trials for their patients and experience a more efficient study initiation process.

The CTTP and Bootcamp represent new investigator focused initiatives that complement other CTSA implemented strategies to address research barriers and streamline processes and trial efficiency.

## Supplementary material

For supplementary material accompanying this paper visit https://doi.org/10.1017/cts.2023.587. click here to view supplementary material

## Clinical trial

This work was presented in part during the Clinical Trials/Education Session at NCATS/NIH Program Meeting, December 2, 2021.
