# Supplementary material for: Preparing clinicians to be site investigators in multicenter clinical trials: A training program at an academic medical center
Source: J Clin Transl Sci. 2023 Jul 10;7(1):e167. doi: 10.1017/cts.2023.587 (PMC10425868; doi:10.1017/cts.2023.587)
Supplement: Supplementary file 1 [file S2059866123005873sup001.docx]

**Supplementary Material:**

Clinical Trials Bootcamp Topics


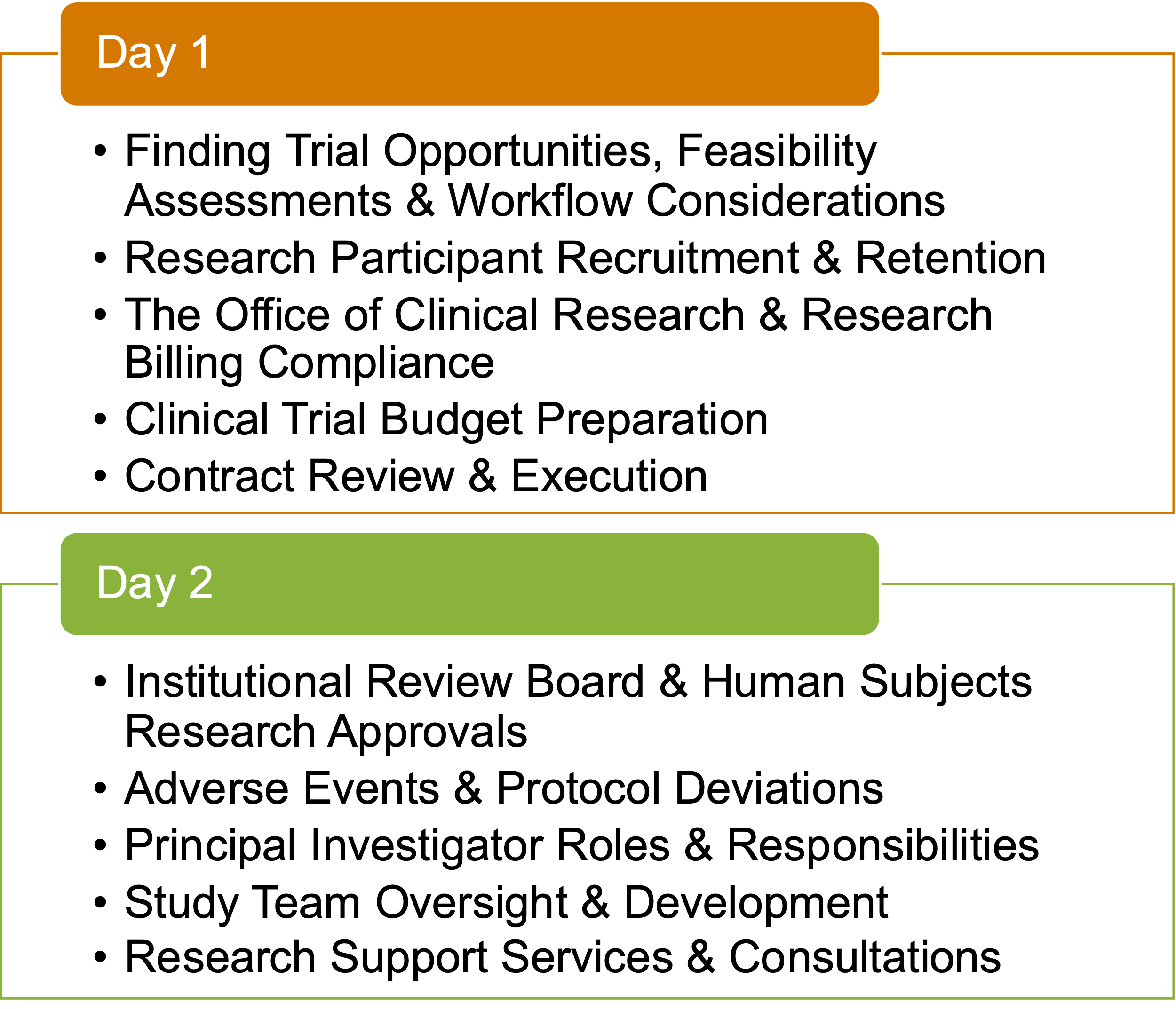


Overall Bootcamp Evaluation

Pre and Post Bootcamp Knowledge Questionnaire
